# Cerium-Doped Bioactive Glass Fibers Attenuate Oxidative Stress and Support Early Liver Regeneration Following Partial Hepatectomy

**DOI:** 10.3390/antiox15101330

**Published:** 2026-10-15

**Authors:** Yanlu Yu, Boyuan Liu, Jing Wu, Ji Wu, Linguo Sheng, Tao Xu, Yuqi Wang, Shuangchi Liu, Roman A. Surmenev, Kai Zheng, Bin Xue

**Affiliations:** 1 Translational Medicine Research Center, Children’s Hospital of Nanjing Medical University, Nanjing 210008, China; yuylyfyy@163.com (Y.Y.); wujing89@njmu.edu.cn (J.W.);; 2 Department of Emergency, Sir Run Run Hospital, Nanjing Medical University, Nanjing 211100, China; 3 Sir Run Run Hospital, Nanjing Medical University, Nanjing 211100, China; 19851778199@163.com; 4 Department of Orthopedics, Sir Run Run Hospital, Nanjing Medical University, Nanjing 211100, China; wujiyfyy@163.com; 5 School of Basic Medical Sciences, Nanjing Medical University, Nanjing 211166, China; 6 Department of Geriatric Medicine, Jiangnan University Medical Center (Wuxi No.2 People’s Hospital), Jiangnan University, Wuxi 214002, China; 7 Research School of Chemistry & Applied Biomedical Sciences, Tomsk Polytechnic University, 634050 Tomsk, Russia; surmenev@tpu.ru; 8 Jiangsu Province Engineering Research Center of Stomatological Translational Medicine & State Key Laboratory Cultivation Base of Research, Prevention and Treatment for Oral Diseases, Nanjing Medical University, Nanjing 210029, China; 9 Department of General Surgery, The Second People’s Hospital of Changzhou, The Third Affiliated Hospital of Nanjing Medical University, Changzhou 213003, China; 10 Collaborative Innovation Center for Cancer Personalized Medicine, Nanjing Medical University, Nanjing 211166, China; 11 Jiangsu Provincial Key Laboratory of Biological Therapy for Organ Failure, Nanjing Medical University, Nanjing 211166, China; 12 State Key Laboratory of Pharmaceutical Biotechnology, Nanjing University, Nanjing 210023, China; 13 NHC Key Laboratory of Antibody Technique, Nanjing Medical University, Nanjing 211166, China; 14 Jiangsu Province Engineering Research Center of Antibody Drug, Nanjing 211166, China

**Keywords:** cerium-doped bioactive glass, oxidative stress, reactive oxygen species, liver regeneration, partial hepatectomy, antioxidant biomaterials

## Abstract

Partial hepatectomy (PH) causes bleeding and oxidative stress, which can slow early liver recovery. To address this, we made cerium-doped bioactive glass fibers (Ce-BGFs) using sol–gel electrospinning. We tested these fibers both in vitro and in a 70% PH mouse model. The fibers showed sustained ion release and lowered H_2_O_2_-induced ROS in hepatocytes. In vivo, Ce-BGFs lowered hepatic ROS fluorescence on postoperative day 1 compared to untreated PH controls (4.17 ± 1.20 vs. 34.52 ± 1.47). Serum AST levels decreased (66.42 ± 15.93 vs. 89.62 ± 16.66 U/L). Ce-BGFs also reduced blood loss (0.039 ± 0.016 vs. 0.102 ± 0.038 g) and bleeding times (13.67 ± 3.21 vs. 37.00 ± 1.73 s). By day 3, we observed more Ki67-positive cells, suggesting better early hepatocyte proliferation (3.92 ± 1.25% vs. 1.14 ± 0.29%). However, liver-to-body weight ratios were not significantly different at this stage. Proteomic profiling showed enrichment in pathways for oxidative stress, inflammation, and pentose phosphate metabolism. Ce-BGF treatment also led to time-dependent cytokine changes and helped preserve liver glycogen after PH. In addition, the PAS-positive area at postoperative day 1 was higher with Ce-BGF treatment than with untreated PH (10.27 ± 1.31% vs. 3.50 ± 1.03%), indicating greater hepatic glycogen preservation. These results show that Ce-BGFs provide local hemostasis and are associated with reduced oxidative stress, attenuated liver injury, enhanced early hepatocyte proliferation, and greater glycogen preservation after PH.

## 1. Introduction

Partial hepatectomy (PH) remains the main treatment for primary liver malignancies, metastatic liver tumors, and selected benign liver diseases [1]. Advances in surgical techniques, perioperative management, and anesthesia has made liver resection much safer overall [2,3]. Nevertheless, postoperative liver dysfunction continues to be a major clinical challenge, particularly after extensive hepatectomy. Acute liver injury, excessive intraoperative bleeding, insufficient future liver remnant (FLR), and post-hepatectomy liver failure (PHLF) remain important causes of postoperative morbidity and mortality [4]. Improving the quality of early liver repair after surgery therefore remains a major objective in hepatobiliary surgery.

Oxidative stress is prominent in the early phase after PH. Surgery, ischemia–reperfusion injury, and hemorrhage can rapidly increase reactive oxygen species (ROS) at the resection margin and disturb local redox homeostasis [5,6]. Excessive ROS directly damage hepatocytes, amplify inflammatory responses, alter intracellular metabolism, and delay the activation of regenerative signaling pathways [5,6]. Limiting ROS burden in the immediate postoperative window may support more efficient liver regeneration.

Current clinical approaches focus mainly on bleeding control and support care [7,8,9]. Topical hemostasis, such as oxidized regenerated cellulose and gelatin sponges, acts mostly by physical tamponade and does little to moderate the oxidative and inflammatory environment at the resection site [10]. Systemic hepatoprotective or antioxidant agents, including N-acetylcysteine, are limited by a short half-life, dose-dependent adverse effects, and poor local accumulation [8]. Experimental studies have explored other antioxidants for PH. Edaravone has been reported to reduce ischemia–reperfusion injury in the remnant liver after PH [11], and thymoquinone, the major bioactive component of Nigella sativa, has been reported to have a hepatoprotective effect following PH under ischemia–reperfusion [12]. In earlier studies, the prevention of the microalga Desmodesmus armatus has been shown to reduce oxidative stress during liver regeneration following PH under acetaminophen-induced toxic injury [13]. Thus, a postoperative material with rapid hemostasis and local antioxidant activity would be useful.

Bioactive materials are increasingly designed to do more than cover wounds passively; they can also influence tissue repair microenvironments [14]. Silicate-based bioactive glasses (BGs) are of interest because of their biocompatibility, controllable ion release, and ability to support inflammation regulation, angiogenesis, and tissue remodeling [15]. Introducing redox-active elements adds the possibility of directly modulating oxidative stress.

Cerium is a redox-active element with SOD- and catalase-like activity scavenging excess ROS [16]. Cerium-containing biomaterials have been shown to reduce oxidative injury and modulate inflammation in several repair settings [17,18]. Notably, Córdoba-Jover et al. reported that cerium oxide nanoparticles enhanced hepatocellular proliferation and liver regeneration in experimental PH [19]. The use of cerium-containing biomaterials directly at the hepatocellular surface to combine local redox modulation with hemostasis remains unexplored.

In this study, we developed cerium-doped bioactive glass fibers (Ce-BGFs) using a sol–gel electrospinning technique. We hypothesized that Ce-BGFs would provide rapid hemostasis while modulating postoperative oxidative stress through sustained releasing bioactive ions. We evaluated oxidative stress, inflammation, metabolic remodeling and early hepatocyte proliferative response using a murine 70% partial hepatectomy model.

## 2. Materials and Methods

### 2.1. Preparation and Characterization of Ce-BGFs

Ce-BGFs were prepared using sol–gel electrospinning as described previously [20,21], with cerium-free BGFs prepared using the same procedure as a material control. Briefly, tetraethyl orthosilicate (TEOS; Shanghai Macklin Biochemical Co., Ltd., Shanghai, China) was hydrolyzed in ethanol (Sinopharm, Beijing, China) /deionized water with 1 M HNO_3_ (Sinopharm, Beijing, China) as the catalyst under continuous stirring. Calcium nitrate (Avantor, Shanghai, China) and cerium nitrate (Sigma-Aldrich, Shanghai, China) were added at the stoichiometric ratios listed in Table 1. After 24 h stirring and 24 h static aging, polyvinyl butyral (PVB; Shanghai Macklin Biochemical Co., Ltd., Shanghai, China; 5% *w*/*v*) was dissolved in the sol to obtain a spinnable solution. Electrospinning was carried out at 15 kV, 0.4–0.57 mL/h, with a 10 cm tip-to-collector distance. As-spun fibers were calcined at 600 °C for 3 h (1 °C/min ramp) to remove the polymer template. Fiber morphology was examined using SEM (JSM-6700, JEOL Ltd., Tokyo, Japan) coupled with EDX. FTIR spectra were recorded in ATR mode (PerkinElmer, Waltham, MA, USA; 4000–600 cm^−1^). Ion release was measured by immersing 20 mg fibers in 10 mL DPBS at 37 °C for up to 7 days; released Si, Ca, and Ce were quantified using ICP-MS (Agilent 7800, Agilent Technologies, Santa Clara, CA, USA).

### 2.2. In Vitro Antioxidant Activity

AML12 murine hepatocytes were cultured in DMEM/F12 medium supplemented with 10% fetal bovine serum and maintained at 37 °C in a humidified incubator with 5% CO_2_. Cells were pre-treated with Ce-BGF extracts (1, 3, and 5 mg/mL) for 24 h, followed by exposure to 200 μM H_2_O_2_. Intracellular reactive oxygen species (ROS) levels were determined using a DCFH-DA fluorescent probe (Beyotime Biotechnology, Shanghai, China) according to the manufacturer’s instructions, and fluorescence intensity was measured using a microplate reader. In addition, the activity of catalase (CAT) was determined using commercial assay kits (Nanjing Jiancheng Bioengineering Institute, Nanjing, China) according to the manufacturer’s protocols.

### 2.3. Hemocompatibility and Coagulation Assessment

Hemocompatibility was evaluated using a 2% RBC suspension in PBS. Materials (1, 3, 5 mg) were incubated with the suspension at 37 °C for 2 h. For this, 0.9% NaCl and distilled water served as negative and positive controls. Hemolysis ratio was calculated from absorbance at 540 nm as follows:

$$Rate\;of\;hemolysis\left(\%\right)=\frac{{OD}_{sample}-{OD}_{NC}}{{OD}_{PC}-{OD}_{NC}}\times 100\%$$

Coagulation was assessed using platelet-poor plasma from citrated whole blood. APTT and PT were measured using commercial kits (Solarbio, Beijing, China) at 37 °C in triplicate.

### 2.4. In Vivo Hemostatic Evaluation

Male C57BL/6J mice were anesthetized, and a standardized 1 cm incision was created on the left liver lobe to establish an acute hepatic bleeding model. Zeolite, BGFs, or Ce-BGFs were immediately applied to the wound surface. Hemostatic time and blood loss were recorded. Blood loss was determined using the gravimetric method. All procedures were performed by the same investigator to minimize experimental variation.

### 2.5. Animal Model

Male C57BL/6J mice (8–10 weeks old, 20–22 g) were obtained from GemPharmatech Co., Ltd. (Nanjing, China) and maintained under specific pathogen-free conditions. All animal procedures were approved by the Animal Ethics Committee of Nanjing Medical University (Approval Code IACUC-2507057). A 70% PH model was established according to the method described by Mitchell et al. After liver resection, sterilized BGF or Ce-BGF membranes were placed directly onto the resection surface before abdominal closure. Sham-operated mice underwent laparotomy without liver resection. Animals were euthanized on postoperative days 1, 3, 5, and 7, and blood and liver tissues were collected for subsequent analyses.

### 2.6. Detection of Hepatic ROS

Fresh liver tissues were embedded in OCT compound and sectioned at a thickness of 10 μm. Sections were incubated with a DCFH-DA fluorescent probe (Kanglang Biotechnology, Shanghai, China) at 37 °C in the dark. Fluorescence images were captured under identical exposure conditions. The fluorescence intensity was quantified using ImageJ software (version 1.53t).

### 2.7. Biochemical Analysis

Serum alanine aminotransferase (ALT) and aspartate aminotransferase (AST) activities were measured using commercial assay kits (Nanjing Jiancheng Bioengineering Institute, Nanjing, China) according to the manufacturer’s instructions.

### 2.8. Cytokine Analysis

Serum concentrations of IL-1β, IL-2, TNF-α, and CCL5 were measured using a multiplex cytokine detection kit (Sai Xiao-Man Biotechnology, Wuhan, China) on an XMplex-100 platform according to the manufacturer’s protocol.

### 2.9. Periodic Acid–Schiff Staining

Periodic acid–Schiff (PAS) staining was performed on 6 μm paraffin sections using a commercial staining kit (Ruiyu Biotechnology, Shanghai, China) following the manufacturer’s protocol.

### 2.10. Cell Proliferation

Ki67 expression was assessed using immunohistochemistry on 6 μm paraffin sections. After antigen retrieval and peroxidase blocking, sections were incubated with rabbit anti-Ki67 (Proteintech Group, Inc., Rosemont, IL, USA; Cat. No.27309-1-AP) overnight at 4 °C, followed by DAB visualization and hematoxylin counterstain.

### 2.11. Quantitative Real-Time PCR

Total RNA was extracted with a commercial kit (Aikerui, Changsha, China). cDNA was synthesized with a reverse transcription kit (Vazyme, Nanjing, China). qPCR was run with SYBR Green on a QuantStudio system (Thermo Fisher Scientific, Waltham, MA, USA). β-actin served as the internal control; relative levels were calculated using 2^−ΔΔCt^. Primer sequences are in Table S1.

### 2.12. Proteomic Analysis

Liver tissues were sent to OE Biotech (Shanghai, China) for quantitative proteomic profiling. The workflow included protein extraction, trypsin digestion, and LC–MS/MS analysis.

### 2.13. Statistical Analysis

Data are presented as mean ± standard deviation (SD). Multiple-group comparisons were performed using one-way ANOVA with appropriate post hoc tests, with Welch’s ANOVA and Dunnett’s T3 test used when variances were unequal. Two-group comparisons were performed using a two-tailed unpaired Welch’s *t*-test. Sample sizes (n) are indicated in the corresponding figure legends. Statistical analyses were performed using GraphPad Prism (version 10.6.0), and *p* < 0.05 was considered statistically significant.

## 3. Results

### 3.1. Fabrication and Characterization of Ce-BGFs

**Figure 1:**
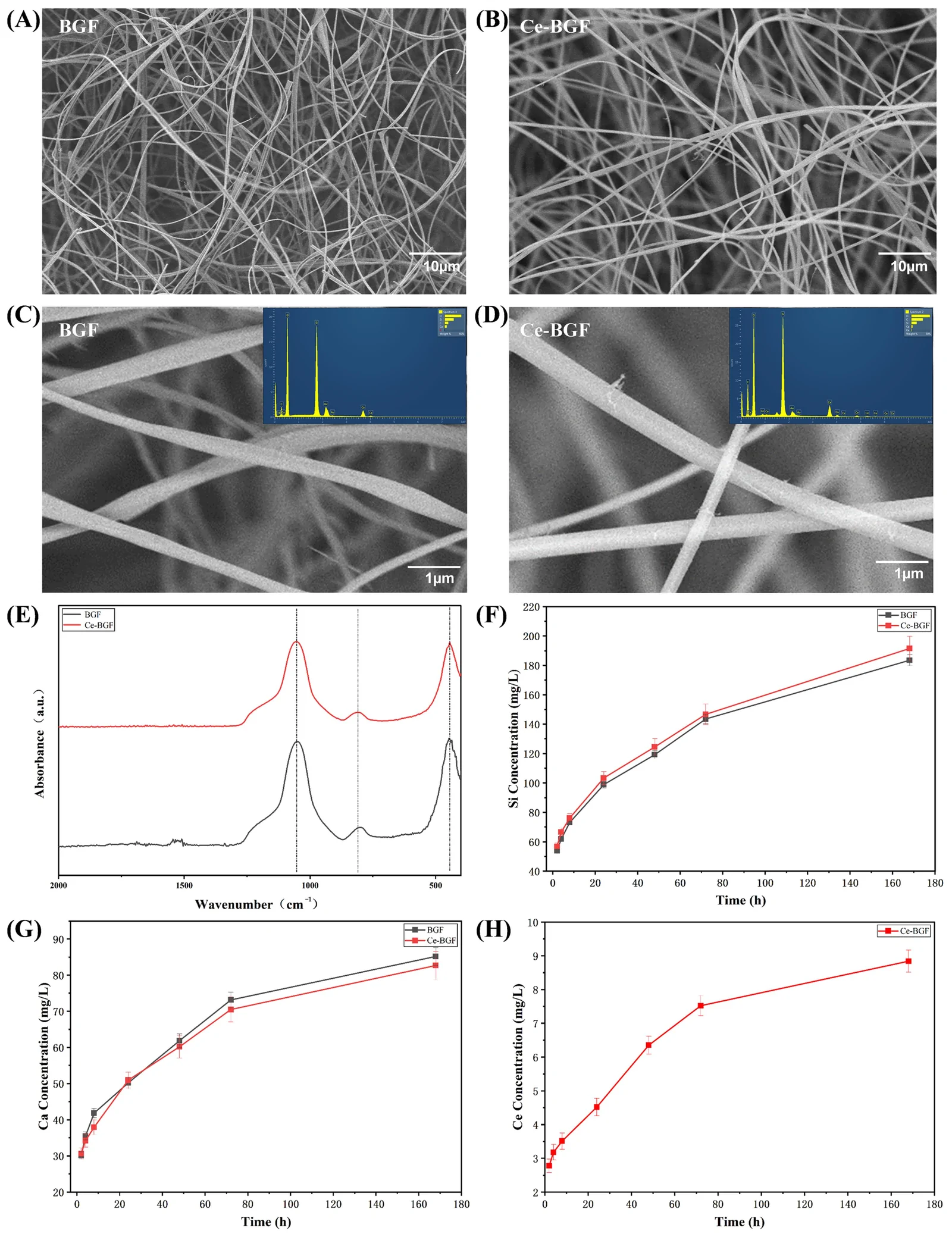
Morphological characterization, elemental composition, chemical structure, and ion-release behavior of BGFs and Ce-BGFs. (**A**,**B**) Representative SEM images of BGFs (**A**) and Ce-BGFs (**B**) showing their continuous fibrous morphologies. Scale bars = 10 μm. (**C**,**D**) Higher-magnification SEM images and corresponding EDS spectra of BGFs (**C**) and Ce-BGFs (**D**), confirming the elemental composition of the fibers and the presence of Ce in Ce-BGFs. Scale bars = 1 μm. (**E**) FTIR spectra of BGFs and Ce-BGFs showing the characteristic vibrational features of the silicate glass network. The vertical dashed lines in (**E**) indicate the characteristic absorption bands of the silicate glass network. (**F**–**H**) Time-dependent release profiles of Si (**F**), Ca (**G**), and Ce (**H**) ions from BGFs and Ce-BGFs.

Ce-BGFs were prepared using sol–gel electrospinning, with BGFs as the cerium-free control. After calcination at 600 °C to remove the polymer template, both BGFs and Ce-BGFs exhibited continuous fibrous morphologies (Figure 1A,B). The observed morphological differences between BGFs and Ce-BGFs may reflect changes in the physicochemical properties of the precursor sol following Ce incorporation, consistent with previous findings for electrospun Ce-doped bioactive glass [17]. EDS analysis confirmed the presence of Ce in Ce-BGFs, whereas no Ce signal was detected in BGFs (Figure 1C,D). FTIR spectra showed characteristic vibrational bands of the silicate network (~445, ~806, and ~1054 cm^−1^) in both materials, with no obvious spectral changes following Ce incorporation (Figure 1E). ICP-MS measurements demonstrated sustained release of Si and Ca from both BGFs and Ce-BGFs over 7 days, while Ce-BGFs additionally exhibited time-dependent Ce ion release (Figure 1F–H). These results confirm the successful incorporation of Ce into the bioactive glass fibers while retaining the characteristic silicate network and demonstrate sustained ion release from Ce-BGFs.

### 3.2. Ce-BGFs Attenuate Oxidative Stress In Vivo and In Vitro

**Figure 2:**
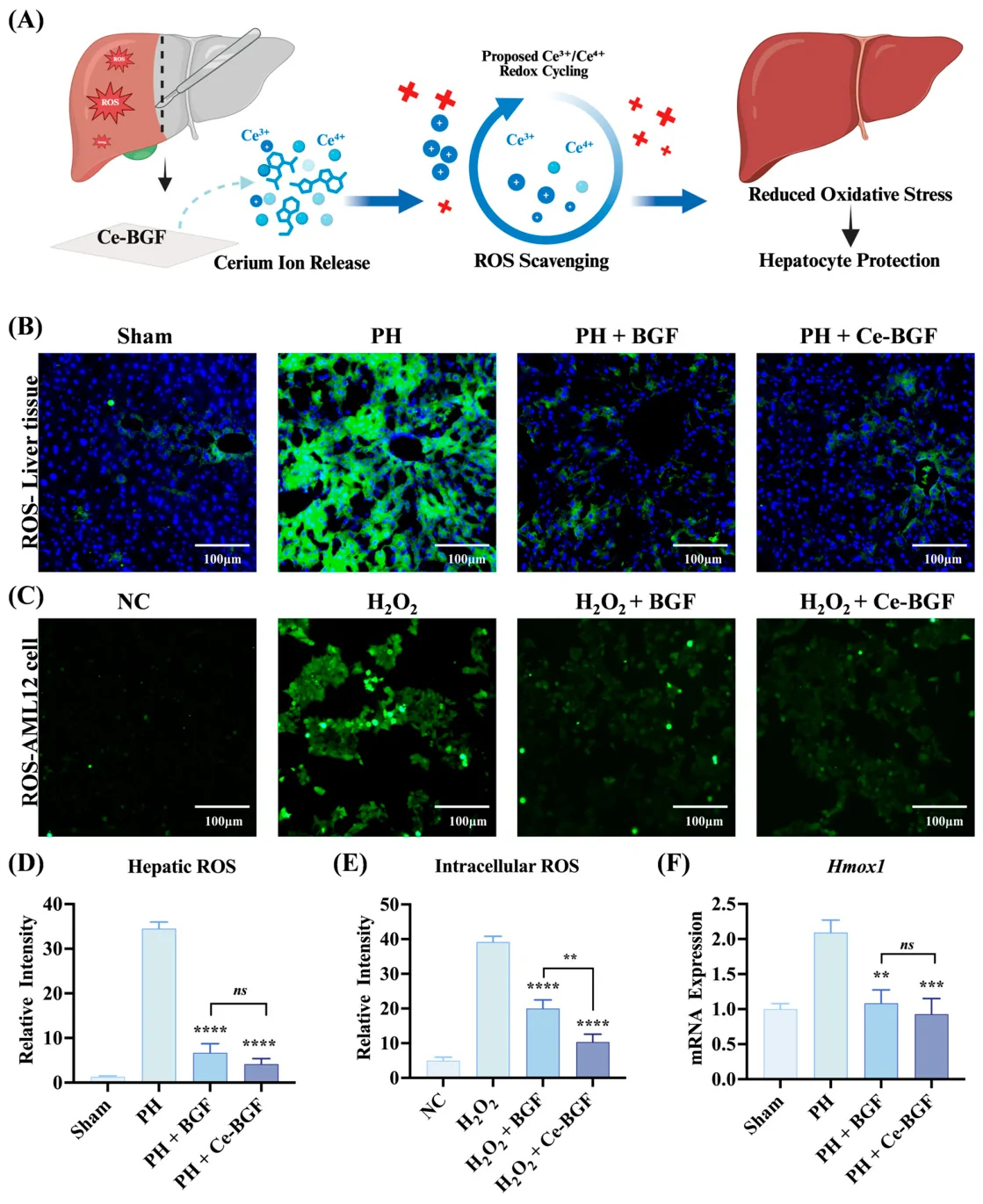
Regulation of oxidative stress by Ce-BGFs in vivo and in vitro. (**A**) Schematic illustration of the proposed antioxidant mechanism of Ce-BGFs, in which cerium ion release and Ce^3+^/Ce^4+^ redox cycling may contribute to reactive oxygen species (ROS) scavenging and attenuation of oxidative stress. In (**A**), red cross-shaped symbols represent reactive oxygen species (ROS), blue circles represent cerium ions, and the circular arrow indicates the proposed Ce^3+^/Ce^4+^ redox cycling. Horizontal arrows indicate the proposed sequence of events. (**B**) Representative fluorescence images of ROS in liver tissues from the Sham, PH, PH + BGF, and PH + Ce-BGF groups on postoperative day 1, detected using DCFH-DA staining. Scale bar = 100 μm. (**C**) Representative fluorescence images of intracellular ROS in AML12 cells under normal control (NC) conditions, following H_2_O_2_ challenge, or following H_2_O_2_ challenge with BGF or Ce-BGF treatment. Scale bar = 100 μm. (**D**) Quantification of hepatic ROS fluorescence intensity on postoperative day 1 (n = 3 mice/group). (**E**) Quantification of intracellular ROS fluorescence intensity in AML12 cells; three replicate wells per condition were analyzed in a single experiment. (**F**) Relative *Hmox1* mRNA expression in liver tissues on postoperative day 1 (n = 7 mice/group). Data are presented as mean ± SD. ** *p* < 0.01, *** *p* < 0.001, and **** *p* < 0.0001; *ns*, not significant.

Surgical trauma and ischemia–reperfusion after PH provoke a rapid ROS burst that can impair hepatocellular integrity [22]. To evaluate the antioxidant effect associated with Ce-BGF treatment, we first examined ROS accumulation in the murine 70% PH model. At postoperative day 1, compared with untreated PH group, Ce-BGFs reduced ROS fluorescence (4.17 ± 1.20 vs. 34.52 ± 1.47; Figure 2B,D). BGF treatment also reduced ROS levels, with no difference between BGFs and Ce-BGFs. Consistent with reduced oxidative burden, the expression of *Hmox1*, which was elevated in the PH group, was lower after Ce-BGF treatment (Figure 2F, Supplementary Figure S1).

We next examined intracellular ROS in AML12 hepatocytes challenged with H_2_O_2_. Pre-treatment with Ce-BGF extracts reduced DCFH-DA fluorescence in a dose-dependent manner (10.34 ± 2.25 vs. 39.18 ± 1.65; Figure 2C,E). Consistent with these findings, Ce-BGF extracts restored CAT activity in H_2_O_2_-treated AML12 cells (Supplementary Figure S2). Together, the in vivo and in vitro findings support an antioxidant effect associated with Ce-BGF treatment.

### 3.3. Hemostatic Performance and Hemocompatibility

**Figure 3:**
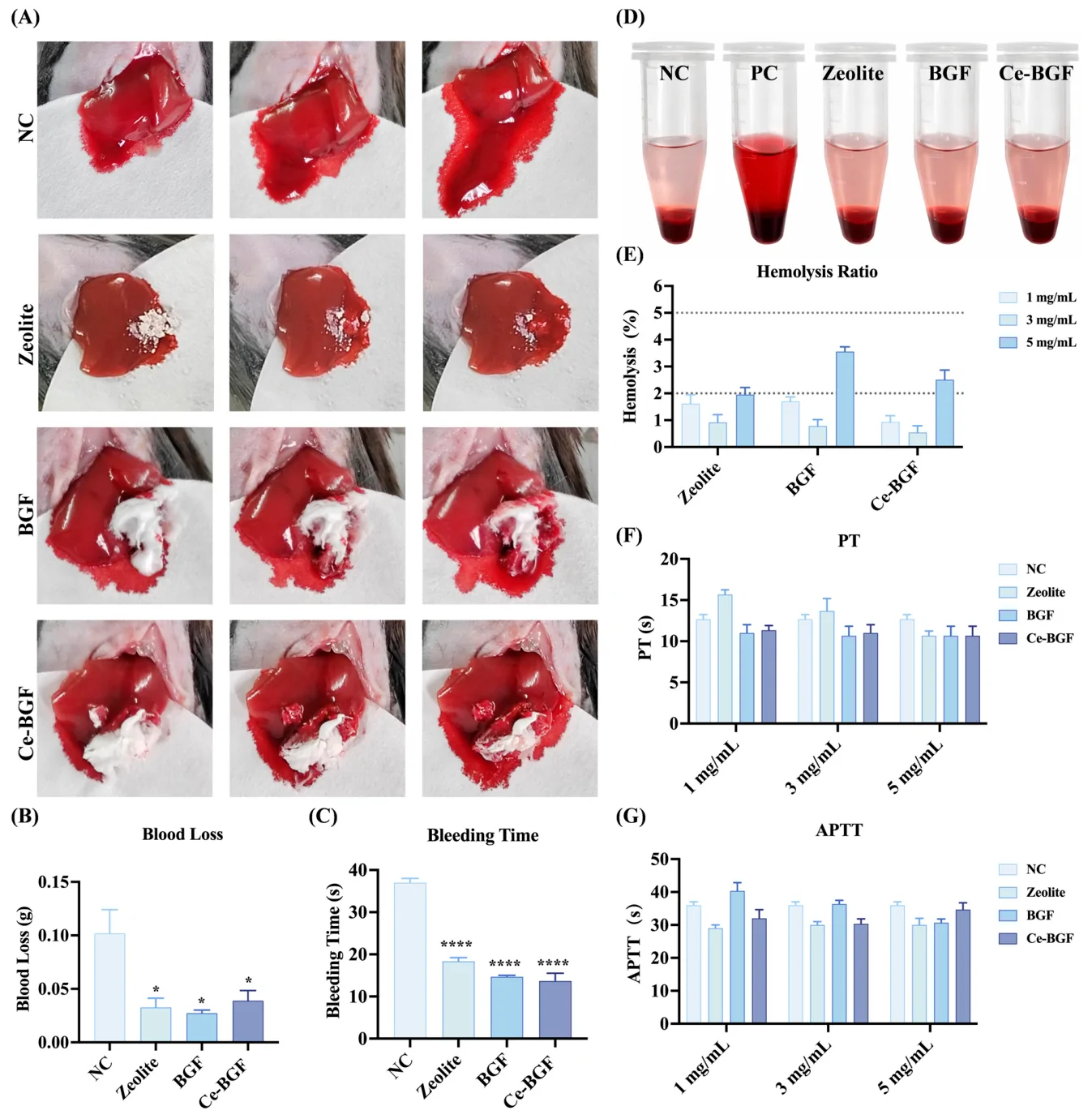
Hemostatic performance and hemocompatibility of BGFs and Ce-BGFs. (**A**) Representative images from the mouse liver hemorrhage model following application of no material (NC), zeolite, BGFs, or Ce-BGFs (n = 3 mice/group). (**B**) Quantification of blood loss and (**C**) bleeding time in the mouse liver hemorrhage model (n = 3 mice/group). (**D**) Representative images of hemolysis assay with human blood, with 0.9% NaCl and double-distilled water serving as the negative control (NC) and positive control (PC), respectively. (**E**) Hemolysis ratios following incubation with zeolite, BGFs, or Ce-BGFs at concentrations of 1, 3, and 5 mg/mL. (**F**,**G**) Prothrombin time (PT; (**F**)) and activated partial thromboplastin time (APTT; (**G**)) following incubation with indicated materials at concentrations of 1, 3, 5 mg/mL. Hemolysis, PT, and APTT assays were performed using blood obtained from a single healthy donor, with replicate measurements performed under each experimental condition. Data are presented as mean ± SD. * *p* < 0.05 and **** *p* < 0.0001.

Rapid hemostasis at the cut surface is a practical requirement after PH [23]. In the mouse liver hemorrhage model, compared with the untreated control, zeolite, BGFs, and Ce-BGFs all reduced blood loss (0.033 ± 0.015, 0.027 ± 0.005, and 0.039 ± 0.016 vs. 0.102 ± 0.038 g, respectively) and shortened bleeding time (18.33 ± 1.53, 14.67 ± 0.58, and 13.67 ± 3.21 vs. 37.00 ± 1.73 s, respectively) (Figure 3A–C). The hemostatic effects observed with BGFs and Ce-BGFs were comparable in magnitude to those of the zeolite control. The comparable hemostatic performance of BGFs and Ce-BGFs suggests that the fibrous glass matrix itself contributes substantially to the local hemostatic effect, potentially by providing a physical barrier and concentrating blood components at the wound surface [14]. We further performed exploratory in vitro assays to characterize the interaction of the materials with human blood. Hemolysis ratios of zeolite, BGFs, and Ce-BGFs at concentrations of 1, 3, 5 mg/mL remained below 5% (Figure 3D,E). PT and APTT measurements showed no marked prolongation following incubation with BGFs or Ce-BGFs across the tested concentrations (Figure 3F,G). These in vitro measurements were performed using blood obtained from a single donor and therefore serve as supportive material-characterization data rather than population-level evidence.

### 3.4. Ce-BGFs Alleviate Acute Liver Injury and Enhance Early Hepatocyte Proliferative Responses

**Figure 4:**
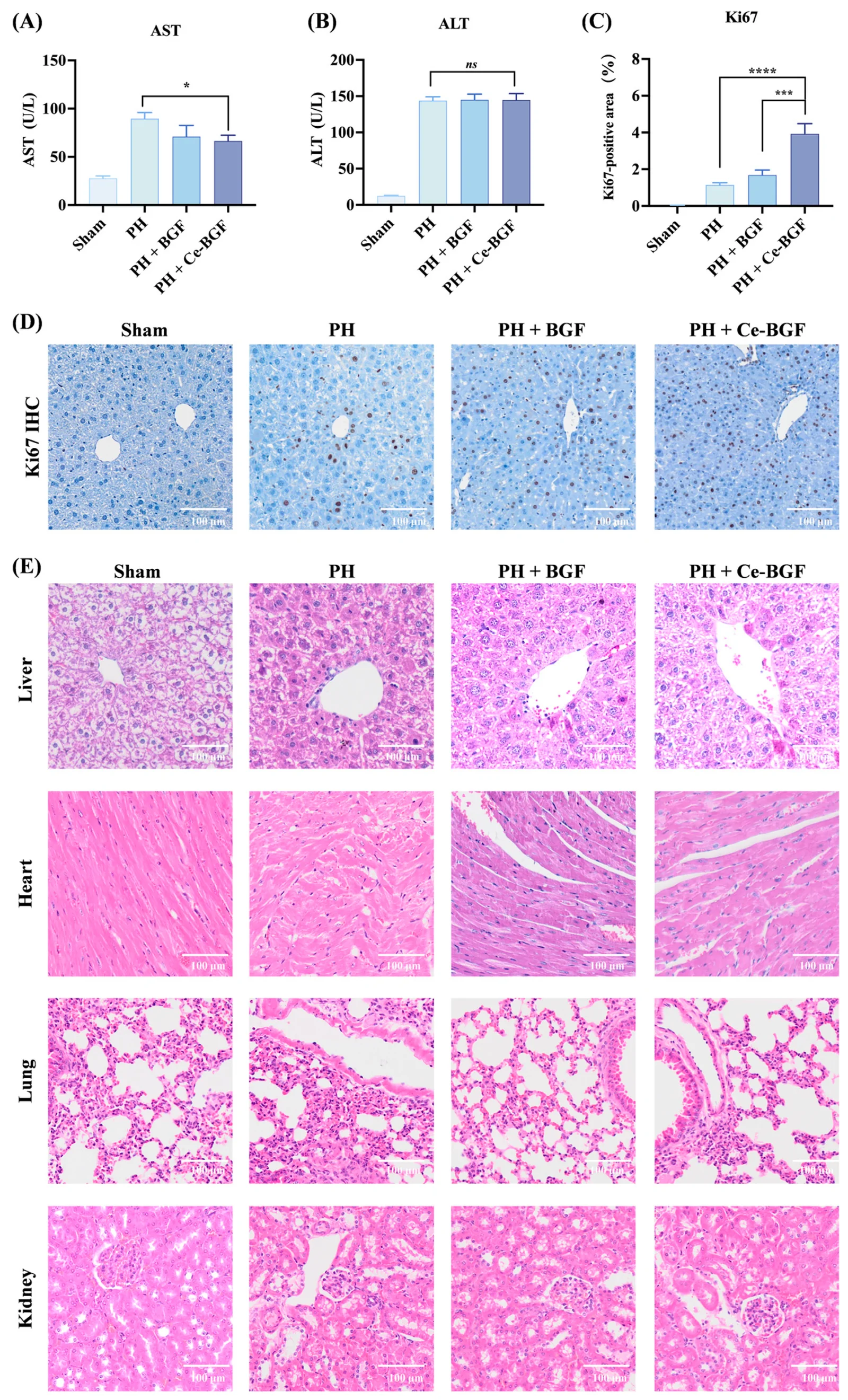
Acute liver injury, early hepatocyte proliferative responses and histological assessment following Ce-BGF treatment. (**A**,**B**) Serum aspartate aminotransferase (AST; (**A**)) and alanine aminotransferase (ALT; (**B**)) levels at postoperative day 1 (n = 7 mice/group). (**C**) Quantification of Ki67-positive area in liver tissues at postoperative day 3 (n = 5 mice/group). (**D**) Representative immunohistochemical images of Ki67 staining in liver sections at postoperative day 3. Scale bar = 100 μm. (**E**) Representative H&E-stained sections of the liver, heart, lung, and kidney at postoperative day 7 (n = 3 mice/group). Scale bar = 100 μm. Data are presented as mean ± SD. * *p* < 0.05, *** *p* < 0.001 and **** *p* < 0.0001; *ns*, not significant.

Serum AST and ALT were markedly elevated in the PH group at day 1. Compared with the untreated PH group, Ce-BGF treatment was associated with a significant reduction in AST (66.42 ± 15.93 vs. 89.62 ± 16.66 U/L), whereas ALT levels did not differ significantly between the PH and PH + Ce-BGF groups (144.63 ± 22.74 vs. 143.74 ± 14.09 U/L) (Figure 4A,B). These findings suggest a partial attenuation of acute hepatocellular injury following Ce-BGF treatment. Ki67 staining at postoperative day 3 showed a significantly greater Ki67-positive area in the PH + Ce-BGF group than in both the PH and PH + BGF groups (3.92 ± 1.25% vs. 1.14 ± 0.29%) (Figure 4C,D), indicating an enhanced early hepatocyte proliferative response. But, liver-to-body weight ratios did not differ significantly among the groups during this early postoperative period (Figure S3), indicating that the increased proliferative responses was not yet accompanied by a measurable increase liver mass. H&E staining of liver, heart, lung, and kidney was performed on postoperative day 7. No overtreatment-associated histopathological abnormalities were observed in the examined tissues of the PH + Ce-BGF group compared with other groups (Figure 4E).

### 3.5. Proteomic Profiling of the Early Post-Hepatectomy Liver

**Figure 5:**
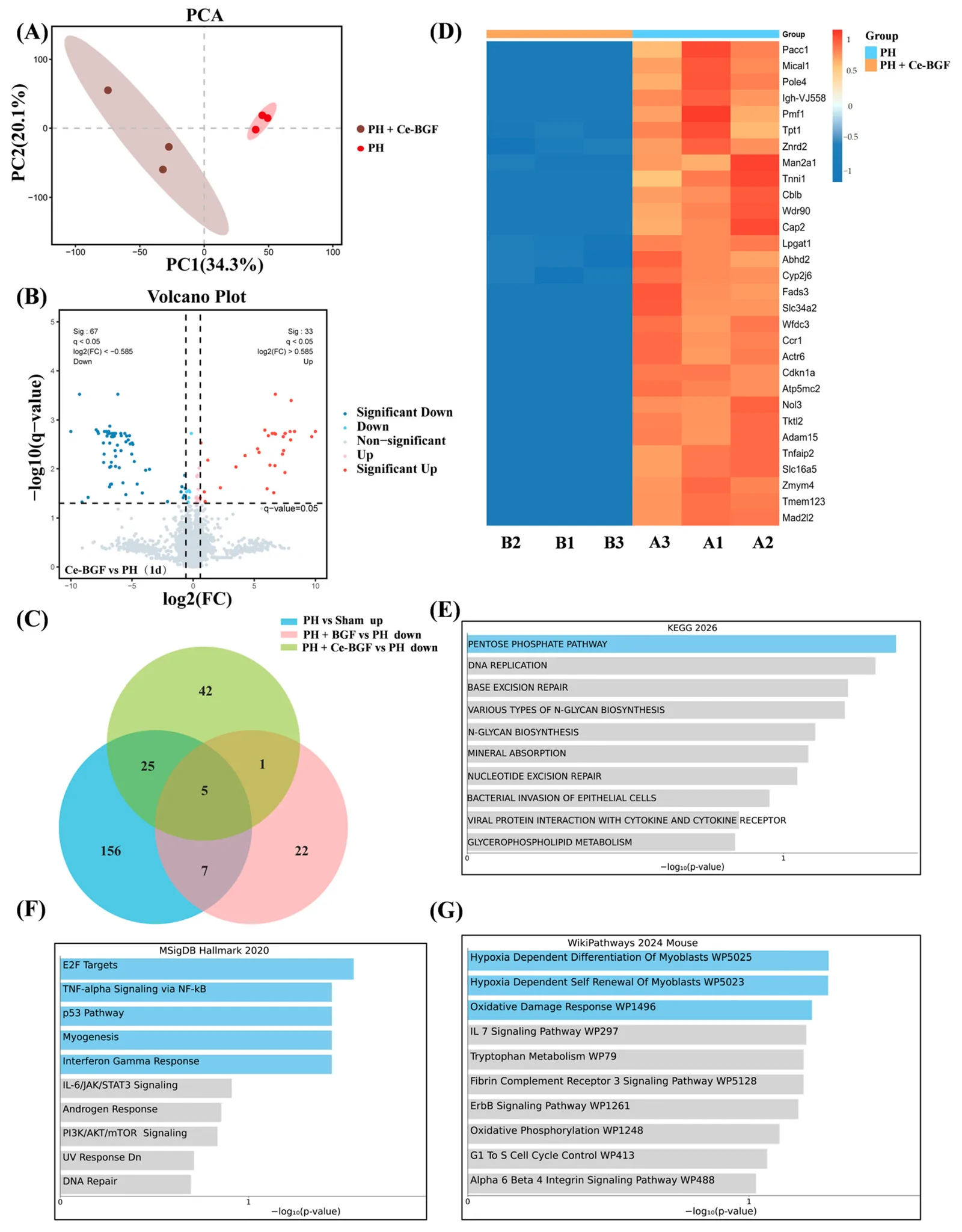
Proteomic profiling of the early postoperative liver. (**A**) Principal component analysis (PCA) of liver proteomic profiles from PH and PH + Ce-BGF groups at postoperative day 1 (n = 3 mice/group). (**B**) Volcano plot showing differentially expressed proteins between the PH and PH + Ce-BGF groups. |log_2_FC| ≥ 0.585 and q < 0.05. The dashed vertical and horizontal lines indicate the fold-change and statistical significance thresholds, respectively. (**C**) Venn diagram comparing proteins upregulated in PH versus Sham with proteins downregulated in PH + BGF versus PH and PH + Ce-BGF versus PH. The 25 proteins uniquely shared between the PH-upregulated and Ce-BGF-downregulated sets, but not the BGF-downregulated set, were selected for subsequent pathway enrichment analyses. (**D**) Hierarchical clustering heatmap of 30 representative differentially expressed proteins between the PH and PH + Ce-BGF groups. (**E**–**G**) Pathway enrichment analyses of differentially expressed proteins (25 Ce-BGF-associated proteins) using KEGG (**E**), Hallmark 2020 (**F**), and WikiPathways 2024 Mouse (**G**). In (**E**–**G**), blue bars highlight selected pathways of interest, whereas gray bars represent the other pathways shown.

Given that ROS, inflammation, and metabolism are intertwined in the early post-PH period [24], we performed global proteomic analysis of liver tissues at postoperative day 1 to explore molecular changes associated with Ce-BGF treatment. PCA showed clear separation between the PH and PH + Ce-BGF groups (Figure 5A). Differential protein expression between these two groups was further visualized using volcano plot analysis (Figure 5B), and hierarchical clustering of 30 representative differentially expressed proteins showed distinct expression patterns between the two groups (Figure 5D). To further identify protein alterations associated with Ce-BGF treatment, proteins upregulated in PH versus Sham were compared with those downregulated in PH + BGF versus PH and PH + Ce-BGF versus PH. Venn diagram analysis identified 25 proteins that were upregulated following PH and downregulated following Ce-BGF treatment but were not included in the BGF-associated downregulated protein set (Figure 5C). Pathway enrichment analyses of these 25 proteins were related to oxidative stress, pentose phosphate metabolism, and inflammatory responses (Figure 5E–G). Notably, they were related to the pentose phosphate pathway (PPP), which is closely linked to cellular redox metabolism and NADPH generation under oxidative stress [25]. These findings guided the subsequent analyses of inflammatory and metabolic responses.

### 3.6. Postoperative Inflammatory Cytokine Kinetics

**Figure 6:**
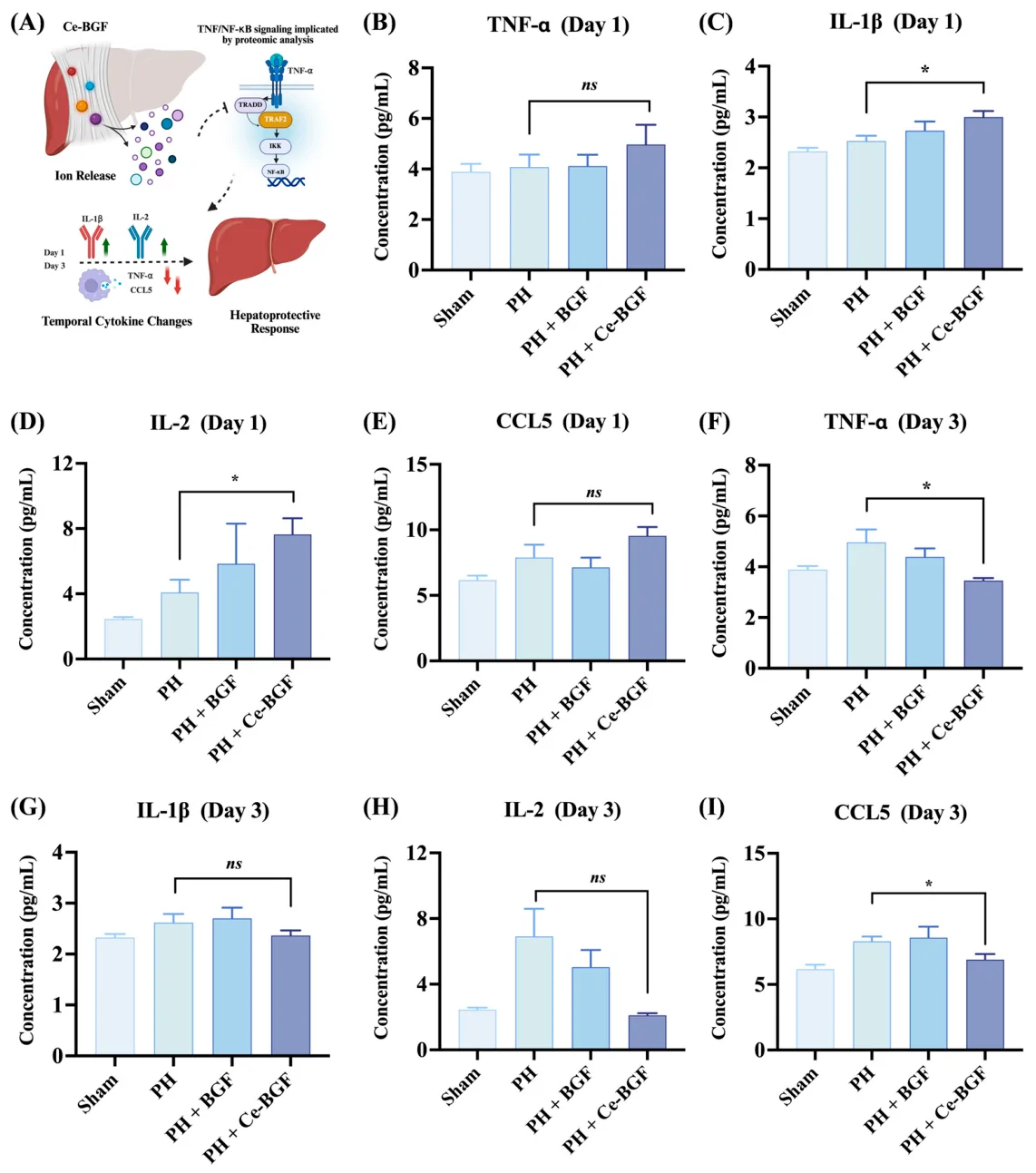
Time-dependent changes in circulating inflammatory cytokines following Ce-BGF treatment. (**A**) Schematic illustration of the proposed temporal pattern of inflammatory cytokine changes associated with Ce-BGF treatment during the early postoperative period. In (**A**), colored circles represent ions released from Ce-BGFs, dashed arrows indicate proposed biological associations, and colored arrows indicate the direction of cytokine changes at different postoperative time points. The TNF-α/NF-κB signaling pathway is illustrated as a potential mechanism suggested by proteomic analysis. (**B**–**E**) Serum concentrations of TNF-α (**B**), IL-1β (**C**), IL-2 (**D**), and CCL5 (**E**) at postoperative day 1 (n = 5 mice/group). (**F**–**I**) Serum concentrations of TNF-α (**F**), IL-1β (**G**), IL-2 (**H**), and CCL5 (**I**) (n = 5 mice/group) At postoperative day 3. Data are presented as mean ± SD. Comparisons shown in the figure were performed between the PH and PH + Ce-BGF groups using two-tailed unpaired Welch’s *t*-tests. * *p* < 0.05; *ns*, not significant.

Guided by the proteomic enrichment results, which implicated inflammatory response pathways, we further examined circulating inflammatory cytokines at postoperative days 1 and 3. At day 1, serum IL-1β and IL-2 levels were higher in the PH + Ce-BGF group than in the PH group (3.00 ± 0.27 vs. 2.53 ± 0.21 and 7.64 ± 2.22 vs. 4.08 ± 1.58, respectively), whereas TNF-α and CCL5 levels did not differ significantly between the two groups (Figure 6B–E). At day 3, TNF-α and CCL5 levels were lower in the PH + Ce-BGF group than in the PH group (4.96 ± 1.01 vs. 3.46 ± 0.22 and 6.89 ± 0.95 vs. 8.29 ± 0.73), while IL-1β and IL-2 showed no significant differences (Figure 6F–I). These results reveal a time-dependent pattern of inflammatory cytokine changes associated with Ce-BGF treatment, characterized by higher levels of selected cytokines at day 1 followed by lower levels of TNF-α and CCL5 at day 3. This temporal pattern is consistent with, but does not directly demonstrate, a transition from an early inflammatory response toward subsequent resolution [26].

### 3.7. Ce-BGFs Preserve Hepatic Glycogen Storage After PH

**Figure 7:**
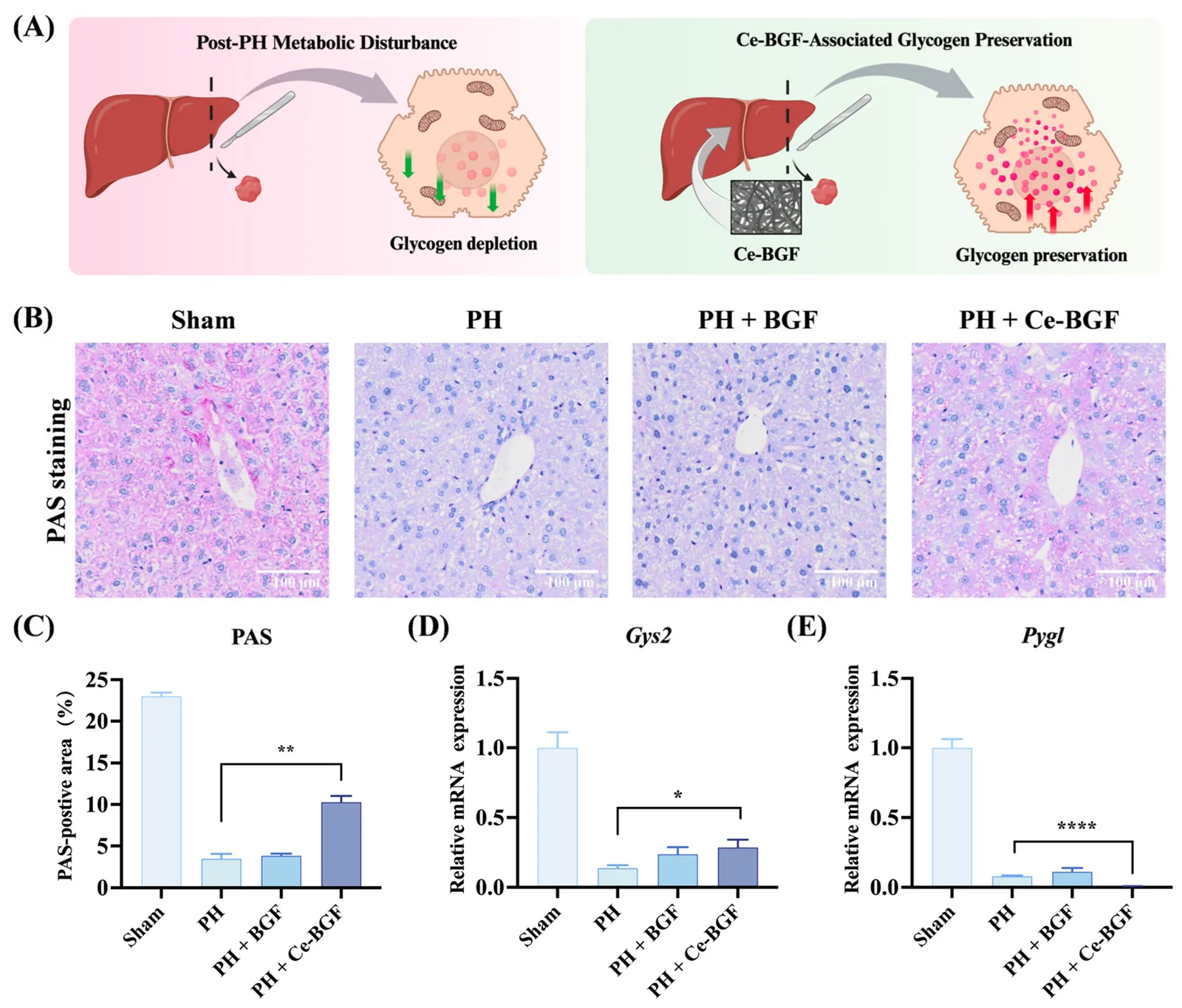
Preservation of hepatic glycogen following Ce-BGF treatment after partial hepatectomy. (**A**) Schematic illustration of post-PH metabolic disturbance and Ce-BGF-associated glycogen preservation. In (**A**), downward and upward arrows indicate glycogen depletion and preservation, respectively, while curved arrows illustrate the proposed sequence of events following partial hepatectomy and Ce-BGF treatment. (**B**) Representative images of periodic acid–Schiff (PAS) staining of liver sections. Scale bar = 100 μm. (**C**) Quantification of the PAS-positive area in liver sections (n = 3 mice/group). (**D**,**E**) Relative hepatic mRNA expression of *Gys2* (**D**) and *Pygl* (**E**) at postoperative day 1 (n = 7 mice/group). Data are presented as mean ± SD. * *p* < 0.05, ** *p* < 0.01, **** *p* < 0.0001; *ns*, not significant.

Consistent with the metabolic pathways identified using proteomic enrichment analysis, PAS staining at postoperative day 1 showed marked glycogen depletion in the PH group, whereas Ce-BGF treatment was associated with greater preservation of hepatic glycogen, as indicated by an increased PAS-positive area compared with the PH group (10.27 ± 1.31% vs. 3.50 ± 1.03%) (Figure 7B,C). In contrast, the BGF group showed only a modest change in PAS staining. At the transcript level, *Gys2*, which encodes glycogen synthase 2, was reduced after PH, but was higher in the PH + Ce-BGF group than in the PH group (0.285 ± 0.149 vs. 0.138 ± 0.056). Conversely, *Pygl*, which encodes liver glycogen phosphorylase, was markedly reduced in the PH + Ce-BGF group compared with the PH group (0.0057 ± 0.0073 vs. 0.079 ± 0.016) (Figure 7D,E).

## 4. Discussion

PH is still the standard treatment for liver disease and certain benign diseases, but postoperative oxidative stress and insufficient future liver remnant prevent functional recovery [27]. Current hemostatic materials control bleeding but do little to address the oxidative microenvironment at the site. In this study, we developed Ce-BGFs as a locally applied fibrous material that both affects hemostatic performance and modulates postoperative oxidative stress. Ce-BGFs reduced ROS accumulation after PH and was associated with a more early hepatocyte proliferative response, and changes in inflammation and metabolic responses in the early postoperative period.

The physicochemical analysis showed successful Ce incorporation and sustained release (Figure 1). In vitro, Ce-BGF extracts reduced H_2_O_2_-induced ROS accumulation in hepatocytes. In the 70% PH model, Ce-BGF treatment reduced hepatic ROS fluorescence at postoperative day 1, and produced a lower *Hmox1*. These results are consistent with the reported redox-modulating properties of cerium-based materials, which are known to dynamically scavenge ROS by Ce^3+^/Ce^4+^ cycling [28,29]. Notably, ROS also participate in regenerative signaling after PH, suggesting that their biological effects are context- and level-dependent [30].

Córdoba-Jover et al. showed that systemic cerium oxide nanoparticles reduced oxidative stress and increased hepatocyte proliferation after PH [19]. In this study, cerium was incorporated into electrospun bioactive glass fibers (Ce-BGFs) for direct application to the liver resection surface. Electrospun fibrous materials suit local wound management because their porous structure and large surface area facilitate fluid handling and local delivery of bioactive agents [20]. Our previous work showed that Ce-doped bioactive glass forms highly hydrophilic, cotton-like fibrous mats with interconnected pores, good in vitro bioactivity, and cytocompatibility [21]. These features let Ce-BGFs sit on the resection bed to provide hemostasis while releasing Ce sustainably. The result is a practical on-site material for managing the postoperative redox microenvironment. BGF itself reduced ROS accumulation in some experiments and differences between BGFs and Ce-BGFs were not consistently significant. The antioxidant phenotype observed here cannot be attributed solely to cerium incorporation; sustained Ce release may contribute to the overall redox-modulation effect of the Ce-BGF platform.

Translationally, the use of cerium can be applied to the sol–gel electrospinning method used to fabricate bioactive glass fibers and the technical feasibility of electrospun Ce-doped bioactive glass fibers has been demonstrated in our previous work [21]. Local application can also avoid repeated systemic administration while maintaining sustained ion release at the resection surface. Manufacturing cost and cost-effectiveness have not been evaluated in this work.

Hemostasis was comparable between BGFs and Ce-BGFs (Figure 3), indicating that the fibrous structure contributes to local hemostasis. Both materials reduced blood loss and shortened bleeding time, suggesting that the cerium incorporation did not affect the hemostasis of the BGF platform. The exploratory human blood assays further showed low hemolysis and no marked prolongation of PT or APTT under the tested conditions. Because these assays were performed using blood from a single donor, they should be interpreted as supportive material-characterization data rather than population-level evidence of hemocompatibility or a specific coagulation mechanism. Thus, Ce-BGFs retain the local hemostatic properties of the underlying fibrous platform while providing sustained local Ce release.

Ce-BGF treatment caused lower serum AST at day 1, compared to the PH group (Figure 4A,B). These results suggest that the biochemical markers of acute hepatocellular injury have been selectively improved rather than uniformly reduced in liver injury. Excessive ROS damage lipids, proteins and DNA, and activate inflammation that increases parenchymal necrosis [24]. The reduction in oxidative stress may therefore contribute to the observed biochemical changes, although the present data do not establish a direct causal relationship. Ki67 staining showed a significantly greater Ki67-positive area in the Ce-BGF group than in the PH group on postoperative day 3 (Figure 4C,D). The absence of significant between-group differences in the liver-to-body weight ratio during the early postoperative period indicates that the increased Ki67 staining was not accompanied by a detectable between-group difference in this measure of liver mass recovery. Accordingly, these findings should be interpreted as evidence of an early proliferative response rather than accelerated liver regeneration. H&E examination at postoperative day 7 further showed no overt histopathological abnormalities in the examined liver, heart, lung, or kidney tissues of Ce-BGF-treated animals compared with the other groups.

Proteomic analysis on day 1 provided a broad molecular basis for these results (Figure 5). Proteins increased after PH and decreased after Ce-BGF treatment, but were not included in the BGF-associated downregulated set, were richly enriched in pathways related to oxidative stress, inflammation, and pentose phosphate metabolism. PPP is typically engaged in oxidative stress to produce NADPH to fight against antioxidant defense [31,32]. Its enrichment in this study could possibly link metabolically with an altered redox environment and postoperative metabolic adaptation [32]. These results are still related: they identify molecular changes accompanying Ce-BGF treatment but do not establish that cerium directly regulates these pathways or that the identified pathways mediate the subsequent phenotype.

IL-1β and IL-2 levels were higher in the Ce-BGF group than in the PH group on postoperative day 1 but did not differ significantly between these groups on day 3. On day 3, TNF-α and CCL5 levels were lower in the Ce-BGF group than in the PH group (Figure 6). Early inflammation can prime regenerative responses [33], whereas persistent inflammation may interfere with subsequent tissue recovery. The observed temporal pattern is therefore consistent with a transition from an early inflammatory response toward subsequent resolution. However, measurements at only two postoperative time points cannot establish the mechanism or kinetics of inflammatory resolution, and these cytokine changes should be interpreted as an associated temporal pattern rather than direct evidence that Ce-BGFs drive this process.

Glycogen preservation is another interesting finding (Figure 7). PAS staining and transcript data for *Gys2* and *Pygl* showed higher hepatic glycogen preservation in Ce-BGF mice early postoperatively. Because PPP competes for glucose-6-phosphate, changes in glucose utilization under reduced oxidative demand may favor glycogen preservation [25]. This interpretation is consistent with the proteomic and histological findings. Neither metabolic flux through the PPP nor glycogen synthesis was directly measured. Therefore, the relationship between reduced oxidative stress, PPP-related changes, and glycogen preservation remains a plausible metabolic interpretation rather than an experimentally established mechanism.

There are some limitations: BGFs alone show antioxidant activity, so Ce’s specific role is not fully understood. Proteomic data suggest pathways were altered but no causal evidence. In vitro blood tests used one donor, so broader hemocompatibility needs testing. Finally, the use of healthy mice limits the generalizability of these findings to patients with underlying liver disease, such as hepatic steatosis or fibrosis. Further validation in relevant diseased liver models is therefore needed.

## 5. Conclusions

In summary, Ce-BGFs provide local hemostasis and modulated oxidative stress after PH. Ce-BGF treatment reduced hepatic ROS accumulation, was associated with lower AST levels and an enhanced early hepatocyte proliferative response, and preserved hepatic glycogen during the early postoperative period. Proteomic and cytokine analyses revealed treatment-related changes in redox, metabolic, and inflammatory responses, although their causal relationships remain to be established. These results suggest Ce-BGFs can be used as a multifunctional local biomaterial for managing the post-hepatectomy microenvironment and allow for further studies in clinically relevant models of liver resection.

## Figures and Tables

**Table 1:** Nominal compositions (in mol%) and reagents used for BGF and Ce-BGF fabrication.

Label	Nominal Composition (mol%)	TEOS (mL)	C_2_H_5_OH (mL)	HNO_3_ (mL)	H_2_O (mL)	Ca (NO_3_)_2_ 4H_2_O (g)	Ce (NO_3_)_3_ 6H_2_O (g)	PVB (% *w*/*v*)
BGF	80SiO_2_-20CaO	8.2	4.2	1.9	0.3	2.1	-	5
Ce-BGF	80SiO_2_-18CaO-2CeO_2_	8.2	4.2	1.9	0.3	1.63	0.4	5

Abbreviations: BGF, bioactive glass fiber; Ce-BGF, cerium-doped bioactive glass fiber; TEOS, tetraethyl orthosilicate; PVB, polyvinyl butyral.

## Data Availability

The mass spectrometry proteomics data have been deposited to the ProteomeXchange Consortium (https://proteomecentral.proteomexchange.org) via the iProX partner repository [34,35] with the dataset identifier PXD085604.

## Supplementary Materials

The following supporting information can be downloaded at: https://www.mdpi.com/article/10.3390/antiox15101330/s1, Supplementary Methods: Western Blot Analysis; Figure S1: Heme oxygenase-1 (HMOX1) protein expression in liver tissues; Figure S2: Catalase (CAT) activity in different treatment groups; Figure S3: Liver-to-body weight ratio and serum transaminase levels after partial hepatectomy; Table S1: Primer sequences used for quantitative real-time PCR (qRT-PCR).

## Abbreviations

The following abbreviations are used in this manuscript:

APTT, activated partial thromboplastin time; AST, aspartate aminotransferase; ALT, alanine aminotransferase; BGF, bioactive glass fibers; CAT, catalase; CCL5, C-C motif chemokine ligand 5; Ce-BGFs, cerium-doped bioactive glass fibers; DCFH-DA, 2′,7′-dichlorodihydrofluorescein diacetate; EDS, energy-dispersive X-ray spectroscopy; FLR, future liver remnant; FTIR, Fourier-transform infrared spectroscopy; *Gys2*, glycogen synthase 2; H&E, hematoxylin and eosin; H_2_O_2_, hydrogen peroxide; *Hmox1*, heme oxygenase 1; ICP-AES, inductively coupled plasma atomic emission spectroscopy; IL-1β, interleukin-1 beta; IL-2, interleukin-2; PAS, periodic acid–Schiff; PCA, principal component analysis; PH, partial hepatectomy; PHLF, post-hepatectomy liver failure; PPP, pentose phosphate pathway; PT, prothrombin time; *Pygl*, glycogen phosphorylase, liver form; ROS, reactive oxygen species; SEM, scanning electron microscopy; TNF-α, tumor necrosis factor alpha.
