# Supplementary material for: Cerium-Doped Bioactive Glass Fibers Attenuate Oxidative Stress and Support Early Liver Regeneration Following Partial Hepatectomy
Source: Antioxidants (Basel). 2026 Oct 15;15(10):1330. doi: 10.3390/antiox15101330 (PMC13701784; doi:10.3390/antiox15101330)
Supplement: Supplementary file 1 [file antioxidants-15-01330-s001.zip › antioxidants-4577583-supplementary.pdf]

Supplementary materials

1.Supplementary Methods

Western Blot Analysis

Liver tissues were homogenized in RIPA buffer containing protease and phosphatase inhibitors. Protein concentration was measured by BCA assay. Equal amounts of protein (25 μg per lane) were separated by 10% SDS-PAGE and transferred to PVDF membranes. After blocking with 5% non-fat milk in TBST, membranes were incubated overnight at 4 ℃ with primary antibodies against HMOX1 (1:1000) and β-actin (1:5000). HRP-conjugated secondary antibodies (1:5000) were applied for 1 h at room temperature. Bands were detected using ECL substrate and quantified by ImageJ. HMOX1 levels were normalized to β-actin. Expected sizes: HMOX1 ~33 kDa, β-actin ~42 kDa.

2. Supplementary Figures

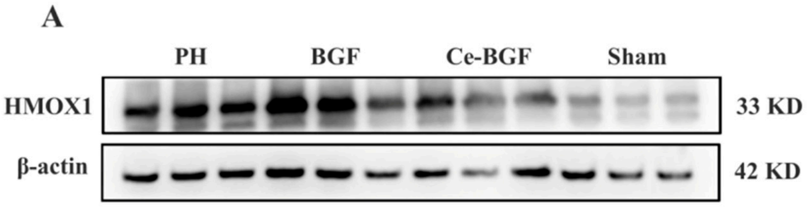

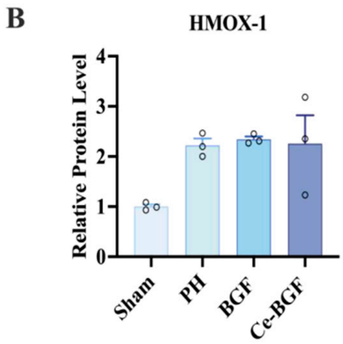

Supplementary Figure S1. Heme oxygenase-1 (HMOX1) protein expression in liver tissues. (A) Representative Western blot bands for HMOX1 and β-actin across Sham, PH, BGF, and Ce-BGF groups. (B) Densitometric quantification of HMOX1 levels normalized to β-actin. Data are

presented as mean±SD with individual data points (n = 3 per group).

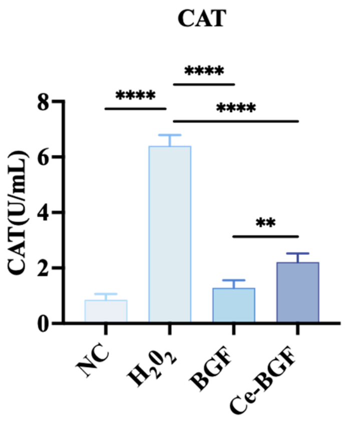

Supplementary Figure S2. Catalase (CAT) activity in different treatment groups. Data are presented as mean±SD (n = 5). ** p < 0.01, **** p < 0.0001, vs. the indicated groups.

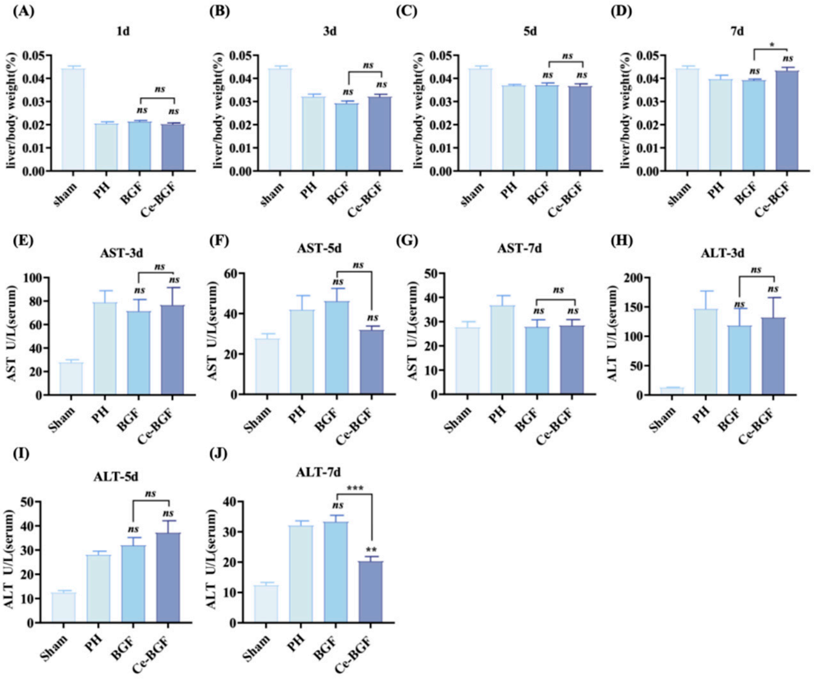

Supplementary Figure S3. Liver-to-body weight ratio and serum transaminase levels after partial hepatectomy. (A-D) Liver-to-body weight ratio (%) at 1, 3, 5, and 7 days post-operation. (E-H) Serum AST levels (U/L) at the corresponding time points. (I－J) Serum ALT levels (U/L) at the indicated time points. Data are presented as mean±SD. Statistical significance was determined

by one-way ANOVA with Tukey's post hoc test. * $p < 0.05$, ** $p < 0.01$,*** $p < 0.001$; ns, not significant.

Supplementary Table S1. Primer sequences use for quantitative real-time PCR (qRT-PCR).

Gene Name	Forward Primer (5'–3')	Reverse Primer (5'–3')
β-Actin	GTGACGTTGACATCCGTAAAGA	GCCGGACTCATCGTACTCC
HMOX1	CCAGAGAAGGCTTTAAGCTGGT	CCAGAGAAGGCTTTAAGCTGGT
GYS2	TCACCACCAACGACGGAT	TCGTTTGAGCTCAGTCAGTTCT
PYGL	ATGTGAAGCGGATCCACGAG	TGATATCCTGGGGCAGCTTTG
